# Primary Adrenocortical Insufficiency Case Series in the Neonatal Period: Genetic Etiologies Are More Common Than Expected

**DOI:** 10.3389/fped.2020.00464

**Published:** 2020-08-12

**Authors:** Jinzhi Gao, Ling Chen

**Affiliations:** Department of Pediatrics, Tongji Hospital, Tongji Medical College, Huazhong University of Science and Technology, Wuhan, China

**Keywords:** primary adrenocortical insufficiency, congenital adrenal hyperplasia, adrenal hypoplasia congenita, familial glucocorticoid deficiency, hyperpigmentation, hyponatremia, hyperkalemia, gene mutation

## Abstract

Primary adrenocortical insufficiency (PAI) is an important cause of morbidity in neonates. The most common cause of PAI in neonates is congenital adrenal hyperplasia (CAH) due to 21-hydroxylase deficiency (21-OHD). Other rarer monogenic cases, for example, adrenal hypoplasia congenita (AHC) or familial glucocorticoid deficiency, also simulate clinical manifestation of 21-OHD, leading to misdiagnosis. The therapies and prognosis of these monogenic cases of PAI are entirely different. This study aimed to compare the differences of clinical data and identify genetic etiologies of PAI cases in the neonatal period. All 7 neonates initially presented with hyperpigmentation, hyponatremia, hyperkalemia, and high serum adrenocorticotropic hormone levels. Only CAH patients showed hyperandrogenism and remarkably elevated serum 17-hydroxyprogesterone levels. All the pathogenic mutations found in CYP21A2 were well known, except c.1069C>T (exon 8). The male patient with AHC had a novel hemizygous deletion of exon 2 in DAX1. The other one with familial glucocorticoid deficiency type 1 had two novel heterozygous mutations in the gene coding melanocortin 2 receptor, c.701C>T (exon 2) and c.119delT (exon 2). Glucocorticoid and/or mineralocorticoid replacement therapy depends on the cause of PAI. Genetic testing can be performed as a alternative diagnostic approach to provide information about therapy, prognosis, and genetic counseling.

## Introduction

The clinical feature of primary adrenocortical insufficiency (PAI) depends on the class of deficient hormone including glucocorticoid, mineralocorticoid, and adrenal androgens, and the extent of hormone deficiency. Glucocorticoid deficiency may cause fatigue, nausea, hypoglycemia, recurrent infections, hyperbilirubinemia, and cholestasis. As a consequence of cortisol deficiency, the production of pro-opiomelanocortin is increased, which is the prohormone of adrenocorticotropic hormone (ACTH) and melanocyte-stimulating hormone. The elevated melanocyte-stimulating hormone results in increased melanin synthesis, causing hyperpigmentation. Mineralocorticoid deficiency may cause failure to thrive, anorexia, dehydration, hypotension, hyponatremia, and hyperkalemia. Andadrenal androgens are associated with sexual development. In general, the above clinical findings of neonates with PAI are non-specific, misdiagnosis and delayed diagnosis are likely to result in potentially life-threatening adrenal crisis. The most common cause of PAI in the neonatal period is congenital adrenal hyperplasia (CAH), which occurs in approximately 1 of 14,200 live births (1, 2). 21-hydroxylase deficiency (21-OHD) caused by mutations in the CYP21A2 gene represents 95–99% of cases of CAH (3). 21-OHD results in the defective conversion of 17-hydroxyprogesterone (17-OHP) to 11-deoxycortisol, which leads to impaired synthesis of cortisol and aldosterone, and increased secretion of adrenocorticotropic hormone (ACTH). ACTH can increase the production of androgens by stimulating adrenal glands. Except for clinical findings caused by glucocorticoid and/or mineralocorticoid deficiency, the increasing androgens may cause clitoridauxe and virilization in female neonates, and hyperpigmentation of scrotum and enlarged phallus in male neonate. According to the chinical presentation determined by residual enzyme activity, CAH is classified into classic forms (salt wasting, simple virilizing) and non-classic form (late onset). Even when the detectable enzyme activity is as low as 1–2 percent, the patient could present with the form of simple virilizing (4).

In the neonatal period, except for 21-OHD, the other rarer genetic disorders including 11β-hydroxylase deficiency, adrenal hypoplasia congenita (AHC) and familial glucocorticoid deficiency (FGD), et al. should also be considered (Table 1). The most common cause of AHC is X-linked genetic defects in the differentiation of adrenocortical cells. The prevalence is 1:140000~1:1200000 (5). The clinical feature of AHC caused by glucocorticoid and mineralocorticoid deficiency is similar to CAH in neonates, but without high androgens levels, along with impaired pubertal development and hypogonadism (6, 7). FGD is a rarer autosomal recessive condition of PAI with a prevalence of < 1:1000000 (8), as it has little more than 50 cases reported in the literature (9). FGD is characterized by unresponsiveness of the adrenal gland to ACTH and preserved aldosterone/renin secretion, so that their clinical presentation is mainly caused by glucocorticoid deficiency (10). The clinical feature of these monogenic cases of PAI are similar, but the therapies and prognosis are entirely different. CAH due to 21-OHD is common, other genetic disorders causing PAI need more attention. In the present study, we conducted genetic analyses of 7 non-consanguineous Chinese children with biochemically characterized PAI. We aim to compare the differences in clinical manifestation and auxiliary examinations, and identify genetic etiologies of PAI cases in the neonatal period, which could contribute to further treatment, prognosis prediction, genetic consultation, and prenatal diagnosis.

**Table 1:** Candidate genes were sequenced to identify mutations in patients with adrenocortical insufficiency.

**Causative gene**	**Name**	**Condition**
NR0B1 (DAX1)	Nuclear receptor subfamily 0 group B member 1	X-linked adrenal hypoplasia congenital
GK	Glycerol kinase 2	Glycerol kinase deficiency
NR5A1(SF- 1)	Nuclear receptor subfamily 5 group A member 1	Adrenal hypoplasia steroidogenic factor-1 deficiency
PRKACA	Protein kinase cAMP-activated catalytic subunit alpha	Cortisol-producing adrenocortical adenomas
DHCR7	7-dehydrocholesterol reductase	Smith- LemliOpitz disease
POMC	Proopiomelanocortin	Signs and symptoms of POMC deficiency (obesity and red hair), high ACTH and low cortisol. Bioinactive but immunoreactive ACTH
PDE8B	Phosphodiesterase 8B	Primary pigmented nodular adrenocortical disease type 3
PDE11A	Phosphodiesterase 11A	Primary pigmented nodular adrenocortical disease type 2
PRKAR1A	Protein kinase cAMP-dependent type I regulatory subunit alpha	Primary pigmented nodular adrenocortical disease type 1
LHX4	LIM homeobox 4	Combined pituitary hormone deficiency type 1
ARMC5	Armadillo repeat containing	Bilateral macronodular adrenal hyperplasia
MC2R	Melanocortin 2 receptor	Familial glucocorticoid deficiency type 1
H6PD	Hexose-6-phosphate dehydrogenase/glucose 1-dehydrogenase	Hydrocortisone reductase deficiency type 1
CDKN1C	Cyclin dependent kinase inhibitor 1C	Intrauterine growth retardation, metaphyseal dysplasia, adrenal insufficiency, genital anomalies
CYP11B1	11-βhydroxylase	11-Beta-hydroxylase deficiency
ABCD1	ATP-binding cassette, subfamily D, member 1	X-linked Adrenoleukodystrophy (X-linked ALD)
SOX3	SRY-box transcription factor 3	X-linked hypopituitarism
GNAS	Guanine nucleotide binding protein, alpha stimulating complex locus	ACTH-independent macronodular adrenal hyperplasia type 1
MRAP	MC2R accessory protein	Familial glucocorticoid deficiency type 2
HSD11B1	Hydroxysteroid 11-beta dehydrogenase 1	Hydrocortisone reductase deficiency type 2
MKS1	MKS transition zone complex subunit 1	Bardet-biedl syndrom type 13
CYP21A2	Cytochrome P450, family 21, subfamily A, polypeptide 2,	21-Hydroxylase deficiency
NNT	Nicotinamide nucleotide transhydrogenase	Nicotinamide nucleotide transhydrogenase deficiency
TBX19	T-box transcription factor 19	ACTH deficiency
MEN1	Multiple endocrine neoplasia 1	multiple endocrine neoplasia type 1
MCM4	Minichromosome maintenance complex component 4	Natural killer cell deficiency, short stature, microcephaly, recurrent viral infections, chromosomal breakage, susceptibility for neoplastic lesions
AIRE	Autoimmune regulator	Chronic mucocutaneous candidiasis, hypoparathyroidism, other autoimmune disorders
CYP17A1	Cytochrome P450, family 17, subfamily A, polypeptide 1	17α-hydroxylase deficiency
NR3C1	Nuclear receptor subfamily 3 group C member 1	Systemic glucocorticoid resistance
PCSK1	Proprotein convertase subtilisin/kexin type 1	Proprotein convertase deficiency type 1
TXNRD2	Thioredoxin reductase 2	Thioredoxin reductase deficiency
CYP11A1	Cytochrome P450, family 11, subfamily A, polypeptide 1	P450 side-chain cleavage enzyme (P450scc)
HESX1	Homeobox gene expressed in ES cells	Pituitary hormone deficiency syndrome type 5
HSD3B2	3-beta hydroxysteroid dehydrogenase 2	3β-hydroxysteroid dehydrogenase type 2
TP53	Tumor protein p53	Adrenocortical carcinoma
CYP11B2	Cytochrome P450 family 11 subfamily B member 2	Congenital hypoaldosteronism
POR	Cytochrome P450, oxidoreductase	P450 oxidoreductase deficiency
PROP1	Paired like homeodomain factor 1	Combined pituitary hormone deficiency type 2
STAR	Steroidogenic acute regulatory protein	Steroidogenic acute regulatory protein (Congenital lipoid adrenal hyperplasia; CLAH)

## Case Presentation

### Subjects

The patients in this study were all from Wuhan, which is located in the center of mainland China. We identified 7 patients who had been diagnosed with PAI in the neonatal period. Patients were not picked up by a newborn screening program but instead were identified by salt-wasting in both males and females. This study was approved by the institutional Human Investigations Committee. Written informed consent was obtained from the legal guardian of each subject.

### Method

Plasma levels of 17-hydroxyprogesterone (17-OHP), testosterone (T), corticotropin-releasing hormone (ACTH), and cortisol were determined at diagnosis by an electrochemiluminescence immunoassay (Roche, Switzerland). The work was conducted at Tongji Hospital affiliated with Tongji Medical College, Huazhong University of Science and Technology, and a commercial genetic testing company (MyGenostics Research Institute). Clinical, laboratory, and genetic studies were undertaken in the 7 patients as well as in their parents. Targeted next-generation sequencing combined with multiplex ligation-dependent probe amplification (MLPA) was used to identify the genetic etiologies of PAI. Candidate genes are shown in Table 1.

## Results

There was no familial relationship among the 7 patients in our report. Each pregnancy was uneventful with term delivery of each patient. All of them presented with hyperpigmentation spreading from head to foot, especially at the genitals and areola, and none of them presented with ambiguous genitalia or intersex conditions. Adrenal imaging, if available, was unremarkable in all cases. Five patients were diagnosed with CAH, one patient was diagnosed with AHC, and the other one was diagnosed with familial glucocorticoid deficiency type 1 (FGD1). Refer to Table 2 for a summary of clinical and biochemical feature.

**Table 2:** The clinical data, mutation spectrum, and laboratory results in the seven consulters diagnosed with PAI.

	**Case 1**	**Case 2**	**Case 3**	**Case 4**	**Case 5**	**Case 6**	**Case 7**
**Diagnosis**	**CAH**	**CAH**	**CAH**	**CAH**	**CAH**	**AHC**	**FGD1**
**Treatment**	**Glucocorticoid** **Mineralocorticoid**	**Glucocorticoid** **Mineralocorticoid**	**Glucocorticoid** **Mineralocorticoid**	**Glucocorticoid** **Mineralocorticoid**	**Glucocorticoid** **Mineralocorticoid**	**Glucocorticoid** **Mineralocorticoid**	**Glucocorticoid**
Age at diagnosis	11d	16d	15d	12d	13d	12d	10d
Sex	Male	Male	Female	Male	Male	Male	Female
Mode of presentation	vomiting, lethargy, hypovolemic shock	pneumonia	vomiting, diarrhea	failure to thrive	jaundice	vomiting	vomiting, lethargy
Gene	CYP21A2	CYP21A2	CYP21A2	CYP21A2	CYP21A2	DAX1	MC2R
Mutation	706_713del8bp(exon3) IVS2-13A/C>G	c.955C>T(exon8) Deletion exon1-4	c.1399_1402del(exon10) hom	Deletion exon1-3 hom	c.1069C>T(exon8) exon1-3 rearrangement	Deletion exon2	c.701C>T(exon2) c.119delT(exon2)
Na (136~145 mmol/L)	127.8	109.1	130.2	115.0	123.7	119.1	128.4
K (3.5~5.1 mmol/L)	5.8	8.3	6.9	10.1	6.6	7.5	5.15
Cortisol (135~650 nmol/L)	100.3	306.5	156.2	122.6	500.3	85	< < 27.6
ACTH (25~100 pg/mL)	>>1250	535.8	575.0	1012	>>1250	>>1250	>>1250
17-OHP (3.2 ± 1.5 nmol/L)	>60	>60	>60	>60	>60	8.13	0.13
Androstenedione (0.66 ± 0.24 nmol/L)	>>35	>>35	>>35	>>35	>>35	11.53	<0.01
DHEA-S* (256 ± 200 nmol/L)	3640	3576	3305	3880	3435	46	46.34
Testosterone (nmol/L)	5.98	9.98	10.01	8.48	7.78	1.69	0.24

*DHEA-S, dehydroepiandrosterone sulfate*.

### Initial Presentation of the Neonates With PAI

The patients came to our attention at the age of 10–16 days with generalized hyperpigmentation. Their manifestations leading to diagnosis with PAI were non-specific. Five patients presented with digestive tract symptoms such as vomiting, diarrhea, and loss of appetite. Two patients presented with pneumonia and jaundice, and electrolyte disturbances were found occasionally. Steroid hormones levels were measured, because the patients presented with the clinical feature of hyperpigmentation and electrolyte disturbance.

### Initial Laboratory Results of the Neonates With PAI

All 7 patients showed hyperkalemia and hyponatremia. Among the 5 patients with CAH, the serum cortisol levels were under normal value in 2 patients and normal in 3 patients, while all the serum ACTH levels were remarkably elevated. Meanwhile, the serum cortisol levels were severely low and ACTH levels were remarkably elevated in patients with AHC and FGD1. Hormonal analyses revealed elevated serum 17-OHP, androstenedione, and dehydroepiandrosterone sulfate (DHEA-S) in patients with CAH, as shown in Table 2. While the male patient with AHC had relatively normal values in serum 17-OHP and androstenedione, lower values in DHEA-S and testosterone. The female patient with FGD1 had an extremely low serum 17-OHP, and low serum androstenedione, DHEA-S, and testosterone values. The mineralocorticoid level in the patient with FGD1 was normal, while her hyponatremia might be due to vomiting, and the extent of hyperkalemia was extremely mild. Hydrocortisone with or without fluorine hydrocortisone therapy with additional saline and glucose infusions were started timely, and the condition was improved markedly.

### Genetic Causes of the Neonates With PAI

Genetic testing in 5 unrelated neonates with CAH all showed mutations in CYP21A2 gene (Table 2). Patient 1 had two heterozygous mutations, 706_713del8bp (exon 3) and IVS2-13A/C>G, inherited from each of his parents. Patient 2 had two heterozygous mutations, including a deletion of exon 1-4 inherited from his mother, and c.955C>T (exon 8) inherited from his father. His mother and father were unaffected carriers. We found a novel homozygous mutation, c.1399_1402del4bp (exon 10), in patient 3. This mutation has not been reported before, and no relevant mutation was found in her parents. Patient 4 had homozygous deletion of exon 1–3, and his patents were both carriers of a deletion of exon 1–3. Patient 5 had two heterozygous mutations. One was c.1069C>T (exon 8) inherited from his father, and the other one was exon 1–3, rearranged from CYP21A2 to a CYP21A1P pseudogene, inherited from his mother.

We found a novel hemizygous deletion of exon 2 in DAX1 in Patient 6 diagnosed with AHC. There were two novel heterozygous mutations, c.701C>T (exon 2) and c.119delT (exon 2) in MC2R gene, in Patient 7 diagnosed with FGD1.

## Discussion

This is a retrospective study of PAI case series. In early infancy, diagnoses of PAI are very easily confused, as all cases present much like each other. This case series focused exclusively on primary adrenocortical insufficiency in neonatal period. The strength of this study was that we were able to obtain genetic testing on all patients.

### Diagnosis and Differential Diagnosis by Clinical Feature and Auxiliary Examinations

The clinical feature of PAI case series is variable but closely similar. As we known, if a patient develops any of the following symptoms: intractable hypoglycemia, hyperpigmentation, hyperkalemia, and hyponatremia with or without circulatory collapse, clitoridauxe and virilization in female neonates, hyperpigmentation of scrotum and enlarged phallus in male neonate, PAI should be considered and steroid hormones should be measured. Isolated hypoaldosteronism could be as first sign of neonates with AHC (7). Patients AHC and CAH often initially present with severe hyperkalemia and hyponatremia, with or without failure to thrive, salt wasting, and vomiting in the neonatal period due to mineralocorticoid deficiency, while patients with FGD do not have salt-wasting. However, transient hyponatremia has been reported in several children with FGD1, sometimes leading to a misdiagnosis of AHC (11). The clinical findings of FGD is very diverse, also including subclinical hypothyroidism, characteristic facial features such as hypertelorism, medial epicanthus and frontal bossing, marginal gross motor developmental delay, gigantism and so on, except for symptoms due to glucocorticoid deficiency (10). Note, though that skin hyperpigmentation is familiar in PAI, absence of obvious hyperpigmentation can not rule out adrenal insufficiency (12). CAH often present with ambiguous or male-appearing external genitalia in girls, while the externalia of patients with AHC or FGD1 often present normal in the neonatal period (6, 13).

Identifying the main laboratory characteristics is helpful for differential diagnosis. 17-OHP, androstenedione, and DHEA-S are substantial elevated in CAH, while all of them are normal or low-level in AHC and FGD1 in the neonatal period. So 17-OHP level is oftern used for neonatal screening for CAH or differential diagnosis between AHC, FGD1, and 21-OHD. Although high serum ACTH and low serum cortisol values are good predictors for PAI, ACTH and cortisol values cannot be used to diagnose PAI alone. That is probably because ACTH and cortisol levels in normal healthy neonates are variable, without a diurnal rythm. Furthermore, the adrenal ultrasound structure characterized by absence or near absence of the permanent zone of the adrenal cortex in patients with AHC. Theoretically, large glands in patients with CAH may be helpful for differentiation. In our report, adrenal ultrasound examination did not demonstrate any apparent abnormality in any of the patients, which may be associated with diagnosis in neonates. Therefore, even if adrenal imaging examination is normal, PAI can not be completely excluded.

### Genetic Causes for PAI Case Series

Although genetic testing for the diagnosis of CAH due to 21-OHD is not essential, it is useful to evaluate borderline cases with equivocal biochemical testing. In order to identify other rarer cases of PAI, such as AHC and FGD (10, 14), and get more information for genetic counseling and prognosis predicting, genetic testing is recommended.

CYP21A2 is located in the human leukocyte antigen class III region on the short arm of chromosome 6p21.3. In this region, CYP21A2 and the CYP21A1P pseudogene are arranged in tandem, and both of them contain ten exons spaced over 3.1 kb. Their nucleotide sequences are 98% identical in their exons and approximately 96% identical in their introns (15). The CYP21A1P pseudogene adds new complexities in detecting mutations in CYP21A2 gene. However, nested PCR sequencing combined with MLPA can detect the actual mutations of CYP21A2 gene effectively. Currently, a general correlation between genotype and phenotype of CAH has been established, but the mutation phenotype does not always correlate precisely the genotype and phenotype (16). The most frequent genetic defect in classic form of CAH is IVS2-13A/C>G (36.1%), followed by deletion of exon 1–3 (19.4%), which both lead to extremely low enzyme activity (17). The combination of mutations IVS2-13A/C>G or c.955C>T (exon 8) with a large deletion has been associated with the most severe salt-wasting phenotype (18–20). The combination of c.955C>T (exon 8) with c.1069C>T (exon 8) has also been associated with salt-wasting phenotype (17). An 8-bp deletion in exon 3, reported as 706_713del8bp in this study, caused a nonsense mutation in exon 3 and was associated with salt wasting (17). The correlation between mutation phenotype and phenotype in our cases was consistent with previous reports. In Chinese patients, the frequencies of IVS2-13A/C>G and c.955C>T are 33.9 and 13.3%, respectively, which are much higher than the frequencies of these mutations in any other population (21).

DAX-1 (dosage-sensitive sex-reversal, on the X-chromosome, gene 1), located on the short arm of chromosome Xp21, also known as NR0B1 gene, consists of two exons with a single 3.4-kb intron. It encodes an orphan member of the nuclear receptor superfamily that lacks the characteristic zinc finger DNA-binding domain. Most studies indicate that DAX-1 acts as a transcriptional repressor of genes involved in the steroidogenic pathway and the development of a functioning hypothalamic-pituitary-gonadal axis (5, 22). Loss of function of DAX1 is associated with adrenal hypoplasia and reproductive dysfunction (22). More than 200 different mutations in the DAX1 gene have been identified to date, but the relationship between mutations and the phenotype of AHC has not been documented (23). The age of onset of adrenal insufficiency, disease severity, and clinical symptoms could vary despite carrying the same mutation (24). It is probable that epigenetic or non-genetic mechanisms could modulate patients' phenotype in X-linked AHC. Frameshift and non-sense mutations may occur within the entire open reading frame, while missense mutations are found mainly in the region encoding the C-terminus of the DAX1 protein (22, 25). Other pathogenic alterations comprise copy-number variations (CNVs) of different sizes (26). In patient 6, AHC was caused by a pathogenic deletion encompassing the entire exon 2 of DAX1, which has not been reported before. This hemizygous deletion was inherited from his mother. His mother is an unaffected carrier. Although we did not confirm the presence of mutant protein, the mutation may result in the most truncated inactive protein so far reported, except for one patient's absence of the entire DAX1 gene sequence region (27).

Causal mutations of FGD have been identified in many genes, inactivating mutations in the ACTH Receptor gene (melanocortin 2 receptor, MC2R) on chromosome 18p11.2 account for about 25% of FGD cases and cause FGD1 (28). MC2R is a protein consisting of 297 amino acids, which has high affinity for ACTH (10). The genetic mutation in MC2R gene may induce trafficking failure of the receptor from the endoplasmic reticulum to the cell surface, and ineffective binding to ACTH (10). Severe or homozygous truncating mutations in the MC2R gene may disturb the renin-angiotensin-aldosterone axis mildly. Such patients need temporary replacement of mineralocorticoid, but this condition does not cause long-term mineralocorticoid deficiency after the intervention is stopped. A little more than 30 mutations have been reported in literatures (8). One mutation, c.701C>T, in patient 7, which was inherited from her mother, results in an exchange of a proline for a leucine. Another 1-bp deletion, c.119delT, in patient 7, which was inherited from her father, results in a shift of the open reading frame. Her parents were both unaffected carriers. These two novel mutations have not been reported and are low-frequency variants in the normal population database. According to American College of Medical Genetics and Genomics (ACMG) guidelines, the clinical significance of either c.701C>T or c.119delT is uncertain but is likely pathogenic. C.701C>T has been predicted to be deleterious, and c.119delT has been predicted to have uncertain effects, by bioinformatics software (SIFT, PolyPhen_2, MutationTaster, GERP++, REVEL).

### The Therapy and Prognosis of PAI Case Series

In all neonates suspected with PAI, glucocorticoid should be initiated to prevent the potentially life-threatening adrenal crisis. If a neonate with PAI presents in adrenal crisis, the confirmatory blood sample is supposed to be obtained first, and then urgent stress doses of hydrocortisone is essential to be supplied. Until the patient is stable and feeding normally, oral glucocorticoid replacement can be chose, and wheather mineralocorticoid replacement is necessary depends on the cause of PAI. Mineralocorticoid replacement is necessary for classic forms of CAH and AHC, not necessary for FGD (29–31). The goal of therapy is to replace deficient steroids and minimize iatrogenic glucocorticoid excess. It is important to use the lowest effective glucocorticoid dose because excessive dosing is associated with poor long-term health outcomes. Patients with CAH should also be monitored for signs of pubertal onset, patients with AHC be monitored for other coexisting endocrine disturbances such as delayed/precocious puberty and infertility (30–33). Because marginal gross motor developmental delay and gigantism may exist in FGD1, growth and development should also be monitored during follow-up in patients with FGD1 (28, 31).

## Conclusion

Upon review of our cases, skin hyperpigmentation and serum electrolyte disturbance are important clinical clues to PAI. In the neonatal period, AHC and FGD are rare etiologies, which should be considered in addition to CAH. Serum ACTH, cortisol, 17-OHP, aldosterone, sex hormone levels, and adrenal ultrasound examination are all important potential distinguishing clues. However, PAI is hard to distinguish because of the non-specific and various clinical feature, especially in the neonatal period. Genetic testing should be included as a alternative diagnostic approach to differentiate all these disorders, and provide information about therapy, prognosis and genetic counseling.

## Data Availability Statement

The datasets generated for this study are available on request to the corresponding author.

## Ethics Statement

This study was approved by the institutional Human Investigations Committee. Written informed consent was obtained from the legal guardian of each subject.

## Author Contributions

JG participated in cases clinical management, data collection, performed the literature search, and drafted the manuscript. LC managed the clinical cases, conceived the work and reviewed the manuscript for important intellectual content.

## Conflict of Interest

The authors declare that the research was conducted in the absence of any commercial or financial relationships that could be construed as a potential conflict of interest.
